# Involvement of S-type anion channels in disease resistance against an oomycete pathogen in Arabidopsis seedlings

**DOI:** 10.1080/19420889.2018.1495007

**Published:** 2018-08-10

**Authors:** Takamitsu Kurusu, Daiki Mitsuka, Chikako Yagi, Nobutaka Kitahata, Tomokazu Tsutsui, Takashi Ueda, Yoshiko Yamamoto, Juntaro Negi, Koh Iba, Shigeyuki Betsuyaku, Kazuyuki Kuchitsu

**Affiliations:** a Department of Applied Biological Science, Tokyo University of Science, Noda, Japan; b Imaging Frontier Center, Tokyo University of Science, Noda, Japan; c Division of Cellular Dynamics, National Institute for Basic Biology, Okazaki, Japan; d Department of Biology, Kyushu University, Fukuoka, Japan; e Japan Science and Technology Agency (JST), PRESTO, Kawaguchi, Japan; f Department of Biological Sciences, The University of Tokyo, Bunkyo-ku, Japan

**Keywords:** Anion flux, SLAC/SLAH channel, disease resistance, oomycete, bacteria

## Abstract

Pharmacological indications suggest that anion channel-mediated plasma membrane (PM) anion efflux is crucial in early defense signaling to induce immune responses and programmed cell death in plants. Arabidopsis SLAC1, an S-type anion channel required for stomatal closure, is involved in cryptogein-induced PM Cl^−^ efflux to positively modulate the activation of other ion fluxes, production of reactive oxygen species and a wide range of defense responses including hypersensitive cell death in tobacco BY-2 cells. We here analyzed disease resistance against several pathogens in multiple mutants of the SLAC/SLAH channels of Arabidopsis. Resistance against a biotrophic oomycete *Hyaloperonospora arabidopsidis* Noco2 was significantly enhanced in the *SLAC1*-overexpressing plants than in the wild-type, while that against a bacteria *Pseudomonas syringae* was not affected significantly. Possible regulatory roles of S-type anion channels in plant immunity and disease resistance against bacterial and oomycete pathogens is discussed.

In various types of mammalian cells, activation of plasma membrane (PM) Cl^−^ channels is an early prerequisite to apoptotic events, including cell shrinkage, cytochrome c release, and programmed cell death (PCD) [1,2]. Pharmacological indications suggest the importance of anion release through the PM in the induction of early defense signaling to induce immune responses and PCD in plants [3,4]. However, the underlying molecular basis of anion efflux and anion channel-mediated regulation of immune responses have not yet been clarified.

Several types of anion channels with different voltage dependency, kinetic properties and anion selectivity have been characterized, mostly by electrophysiological techniques in plants [5–7]. Recent molecular and electrophysiological studies showed that Arabidopsis SLAC1 is required for anion channel activity in the PM of guard cells and is more permeable to Cl^−^ than malate [5], indicating that SLAC1 functions as a slow-type (S-type) anion channel located at the PM in plant cells. This channel, activated by the stress hormone abscisic acid (ABA), ozone, and CO_2_, is involved in the early steps leading to the volume regulation of guard cells and stomatal closure [8–10]. Four orthologs of *SLAC1, SLAH1-4* have been identified in Arabidopsis and constitute the SLAC/SLAH family [8]. However, the physiological roles of SLAC/SLAH family proteins in plant immunity have not yet been elucidated.

We recently revealed that Arabidopsis SLAC1 functions in the early signaling events triggered by cryptogein, a proteinaceous elicitor from an oomycete *Phytophthora cryptogea*, to induce PCD in tobacco BY-2 cells. The functional characterization of *SLAC1*-overexpressing lines suggests that SLAC1 mediates cryptogein-induced Cl^−^ efflux through the PM to positively modulate the elicitor-triggered activation of extracellular alkalinization, NADPH oxidase-mediated production of reactive oxygen species (ROS), and a wide range of defense responses including PCD in BY-2 cells [3,4].

In contrast, in the Arabidopsis *SLAC1*-overexpressing plants, ROS production and expression of defense-related genes, *PR1* and *AtRbohD*, triggered by flg22, a microbe-associated molecular pattern (MAMP) from bacteria, was not affected significantly in comparison with the control [4]. These data were consistent with a previous study showing that the membrane potential change triggered by flg22 as well as elf18, another typical bacterial MAMP, was not affected by anion channel inhibitors including DIDS, or by a T-DNA insertional mutation in *SLAC1* or *SLAH3* gene in mesophyll cells [11,12]. These results suggest that the SLAC/SLAH family may not play a major role in MAMP-triggered immunity (PTI) in Arabidopsis. Effects of PM anion efflux or SLAC/SLAH channels in plant disease resistance against pathogens have not been so far studied.

In order to investigate the possible involvement of anion efflux mediated by SLAC/SLAH channels in disease resistance by Arabidopsis to bacterial and oomycete pathogens, *SLAC1*-overexpressor (*SLAC1-OE), slac1/slah3* double-mutant, and the wild-type seedlings were infected with a virulent strain of an obligate biotrophic oomycete *Hyaloperonospora arabidopsidis* (*Hpa* Noco2). As shown in Figure 1(a,b), the spread of conidiophore formation (indicative of oomycete reproduction) was significantly lower in the *SLAC1-OE* than in wild-type Col-0 seedlings (compatible line). Moreover, the frequency of the spread of conidiophores was also lower in the *SLAC1-OE* than in the wild-type (Figure 1(c)), indicating the enhanced resistance against the oomycete at the seedling stages. The conidiophore formation was also slightly restricted in the *slac1/slah3* mutant phenotype (Figure 1(b,c)). Microscopic examinations revealed that fungal hyphal growth monitored by trypan blue staining was observed in all plant lines, except for the resistant line La*-er* (Figure 1(a-c)).

**Figure 1.**
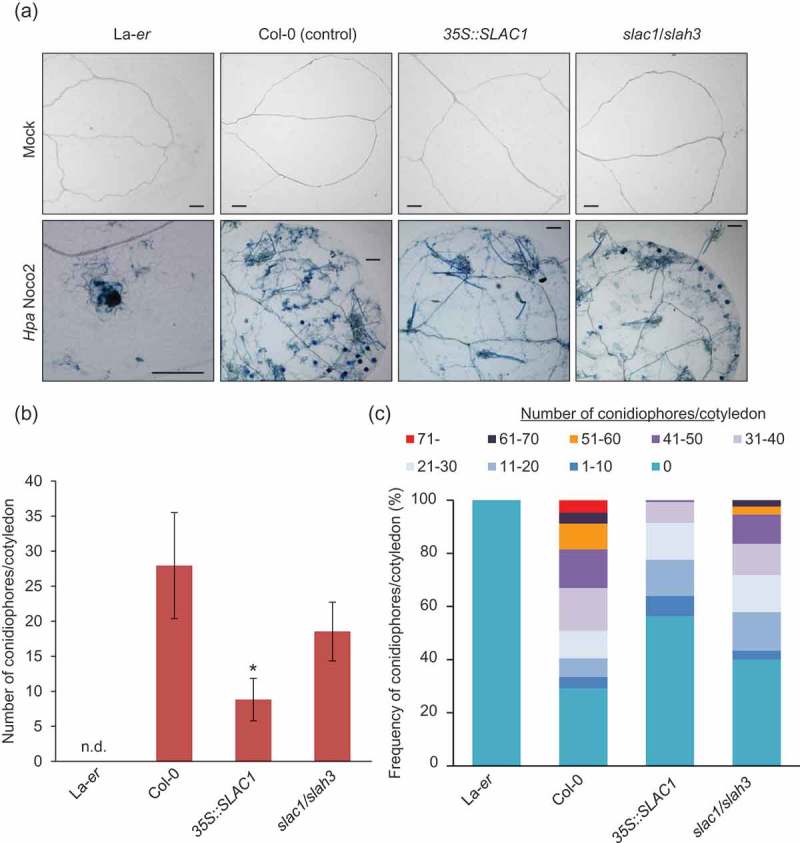
**Depletion of *SLAC1/SLAH3* and overexpression of *SLAC1* result in increased disease resistance against the obligate biotrophic oomycete *Hyaloperonospora arabidopsidis* (*Hpa* Noco2)**. (a) Plant cell death and hyphal growth in representative Arabidopsis plants 7 days after infection (5.0 × 10^4^ spores ml^−1^) as visualized by trypan-blue staining; the resistant La-*er* line were used as a control to display host cell death at infection sites. Scale bar: 100 μm. Data are representative of three experiments. Similar results were obtained in three independent experiments. (b) The number of conidiophores shown in (a) were quantified. Data are mean ± SE; n = 3 independent samples. * *P* < 0.05; significantly different from the control (Col-0). N.d.: not detected. (c) Categorization of conidiophore formation in 2-week-old Arabidopsis plants. Data are representative of three experiments. Similar results were obtained in three independent experiments.

Resistance against biotrophic microbial pathogens is assumed to be predominantly due to the salicylic acid (SA)-dependent mechanisms of resistance [13–15]. Since the *SLAC1-OE* plants were found to be more resistant to the biotrophic oomycete *Hpa* Noco2, we investigated whether SA-dependent defense gene activation was constitutively enhanced in the *SLAC1-OE* plants by analyzing the expression of the SA-inducible marker genes, *PR1, PR2*, and *PR5*. As shown in Figure 2, the transcript levels of these SA marker genes in uninfected seedlings were comparable among the wild-type, *SLAC1-OE* and *slac1/slah3* mutant, suggesting that SA-dependent pathway is not affected by the expression levels of *SLAC/SLAH* channels at least in uninfected seedlings. Either defense signaling responses upon infection of the oomycete or regulation of SA-independent immune responses against an oomycete pathogen may be up-regulated by the overexpression of *SLAC1*.

**Figure 2.**
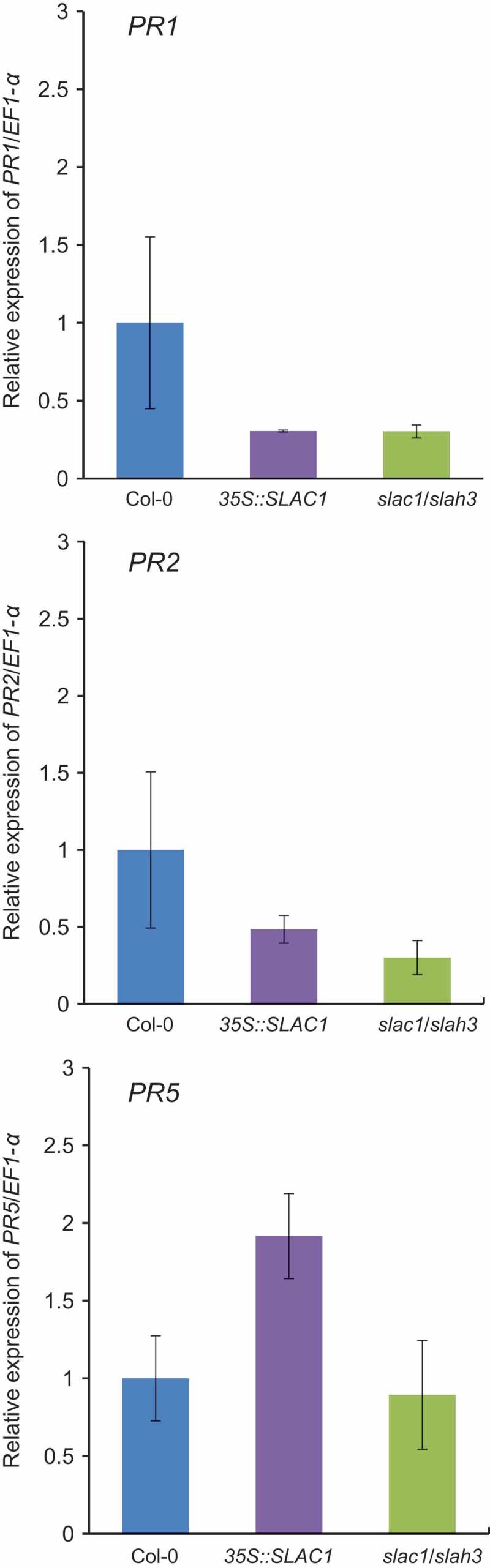
**Effects of expression levels of *SLAC/SLAH* family genes on SA-dependent defense gene activation at the basal level in Arabidopsis**. The expression of SA-dependent defense genes (*PR1, PR2*, and *PR5*) in Arabidopsis leaves. The amount of each mRNA was calculated from the threshold point located in the log-linear range of RT-PCR. Total RNA was isolated from Arabidopsis grown under normal growth conditions. Data are the mean ± SE of three independent experiments.

The present results suggest importance of SLAC/SLAH-mediated anion fluxes in the defense signaling pathway against the oomycete in Arabidopsis seedlings. Resistant phenotypes of the *SLAC1-OE* plants are more critical than that of the *slac1/slah3* mutant (Figure 1(b,c); Supplemental Figure 1), suggesting that SLAC/SLAH-mediated anion fluxes positively modulate plant defense responses against the oomycete *Hpa* Noco2 at least in Arabidopsis seedlings. This hypothesis is consistent with the enhanced defense responses by the ectopic-overexpression of Arabidopsis *SLAC1* in tobacco BY-2 cells triggered by cryptogein [4]. The potential roles of anion fluxes mediated by SLAC/SLAH family in the regulation of disease resistance against *Hpa* Noco2 is an important topic for future research.

It currently remains unclear why the resistance trend against oomycetes was observed in Arabidopsis *slac1/slah3* mutant. SLAC/SLAH family plays important roles in the control of nitrate loading of the root xylem [16]. Limitations in several nutrient sources may be responsible for reduced conidiophore formation in these plants. The limited availability of major plant carbon or nitrogen sources may explain changes in conidiophore formation in the *slac1/slah3* mutant.

To investigate whether SLAC/SLAH channels also affect disease resistance against bacteria pathogens, we infiltrated *slac1/slah3* mutant and *SLAC1-OE* plants with a bacterial pathogen *Pseudomonas syringae*. The overexpression of *SLAC1* and a defect in *SLAC1/SLAH3* genes did not have a significant effect on the growth of the virulent hemibiotrophic bacterial strain *P. syringae* pv. *tomato* DC3000 (*Pst* DC3000) (Figure 3(a)), suggesting limited roles of the SLAC/SLAH channels in basal resistance against a bacteria pathogen. Since PTI plays critical roles in basal resistance [17], this result is consistent with the previous studies showing limited roles of the SLAC/SLAH channels in PTI [4,11]. Growth of *Pst-avrRpt2* bacteria (the avirulent strain) was also comparable between wild-type, *slac1/slah3* mutant, and *SLAC1-OE* plants (Figure 3(b)), suggesting their limited roles in the effector-triggered immunity against the bacterial pathogen.

**Figure 3.**
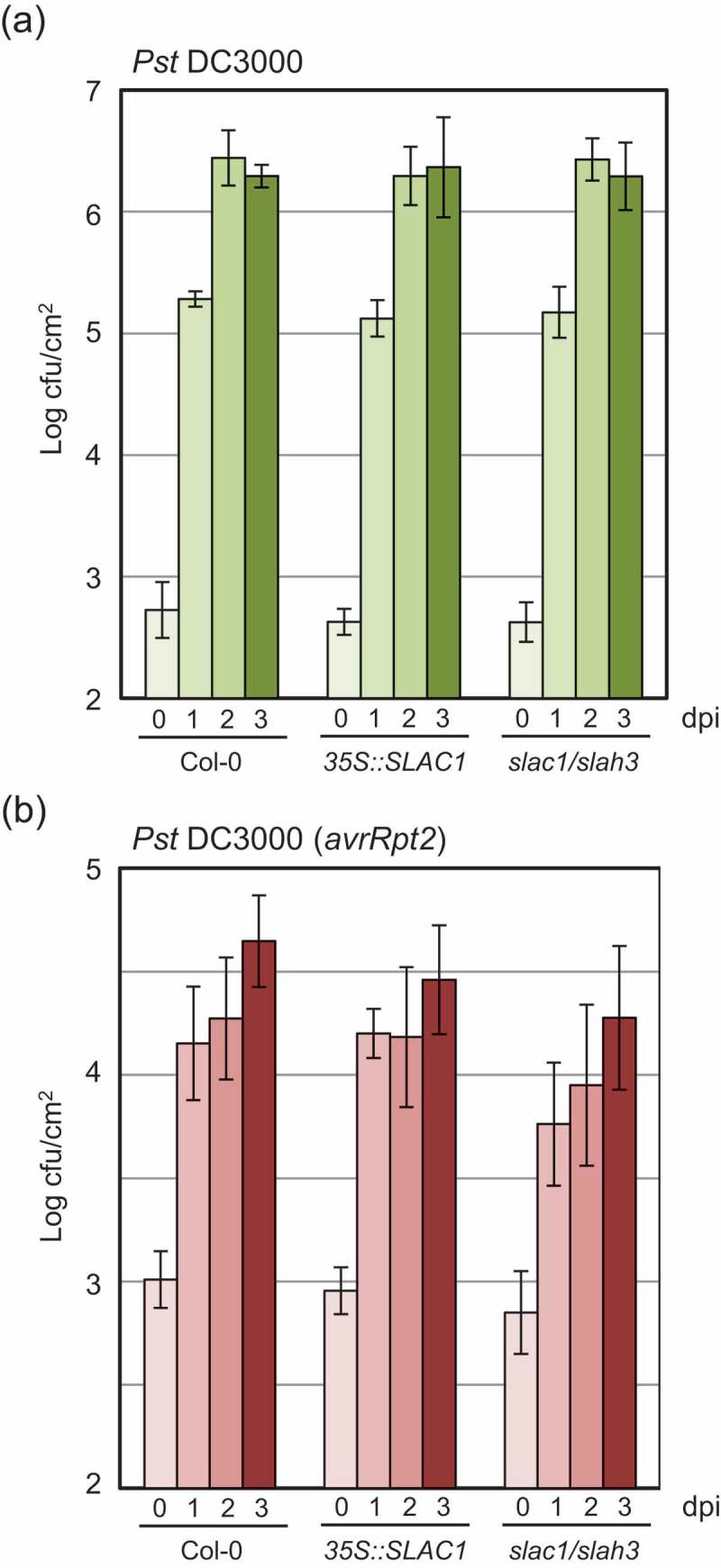
**Effects of expression levels of *SLAC/SLAH* family genes on disease resistance against the bacterial strain *Pseudomonas syringae* in Arabidopsis**. Wild-type Col-0, *slac1/slah3*, and *SLAC1-* overexpressing plants grown under long-day conditions for 3 weeks were injected into plant leaves with a syringe-inoculated with 1.0 × 10^5^ cfu ml^−1^ of virulent *Pseudomonas syringae* pv. *tomato* DC3000 (*Pst* DC3000) as well as the avirulent strain *Pst-avrRpt2*. After 0, 1, 2, and 3 days, bacterial growth was quantified by counting colony-forming units. Results represent means ± SDs (n ≥ 6), and the entire experiment was repeated at least three times with similar results. dpi; days post-inoculation.

Collectively, the present results suggest possible involvement of S-type anion channels in the resistance against the oomycete pathogen, while we did not find significant effects on the resistance against a bacterial pathogen, *Pst* DC3000. In Arabidopsis cultured cells, pharmacological indications suggest that R-type anion channels play key roles in cell death and ROS production triggered by the non-specific plant pathogen *Xanthomonas campestris* [18]. Other anion channel members including the R-type may be functionally redundant in some pathways. Roles of various anion channels may vary among cell types and the triggering signals/types of plant-pathogen interactions.

## Materials and methods

### Plant materials and growth conditions

*slac1-3* (SALK_099139) and *slah3-4* (SALK_111623) seeds were obtained from the Arabidopsis Biological Resource Center. *slac1-3/slah3-4* double mutant was obtained through standard crossing and genotyping in the F2 generation.

Surface-sterilized seeds of *Arabidopsis thaliana* (Col-0, La-*er*) and a *SLAC1* overexpressing plant [4] as well as a *slac1-3/slah3-4* mutant line were germinated on MS medium containing 0.8% agar and grown in a growth chamber under long day conditions (16 h light/8 h darkness, 22°C).

### Real-time RT-PCR quantification

Total RNA was isolated using NucleoSpin RNA Plus kit (TaKaRa, Shiga, Japan) according to the manufacturer’s protocol and quantified with a spectrophotometer. First-strand cDNA was synthesized from 500 ng total RNA using ReverTra Ace qPCR RT Kit (TOYOBO, Osaka, Japan).

Real-time PCR was performed using an ABI PRISM 7300 sequence detection system (Life technologies) with THUNDERBIRD SYBR qPCR Mix (TOYOBO, Osaka, Japan) and gene specific primers. The following PCR primers were used: PR1-RealF, 5ʹ-GTGGGTTAGCGAGAAGGCTA-3ʹ; PR1-RealR, 5ʹ-ACTTTGGCACATCCGAGTCT-3ʹ; PR2-RealF, 5ʹ-AGTCGATAGTCGATGCTAGTG-3ʹ; PR2-RealR, 5ʹ-GTACGTAGCTAAGCATGATC-3ʹ; PR5-RealF, 5ʹ-GTCAGCATGAAACAATGGCA-3ʹ; PR5-RealR, 5ʹ-GCTAGCATAGCTAAAGGCC-3ʹ; AtTUB2-RealF, 5ʹ-ATTCCCCCGTCTTCACTTCT-3ʹ; AtTUB2-RealR, 5ʹ-CACATTCAGCATCTGCTCGT-3ʹ;. Relative mRNA abundances were calculated using the standard curve method and normalized to corresponding *AtTUB2* gene levels. Standard samples of known template amounts were used to quantify PCR products.

### RT-PCR analysis

Total RNA isolation and cDNA synthesis was performed as described above. PCR amplification was performed with an initial denaturation at 95ºC for 3 min followed by the indicated number of cycles of incubations at 94ºC for 30 s, 55ºC for 90 s, and 72ºC for 1 min, and a final extension at 72ºC for 10 min using Arabidopsis *SLAC1*-specific primers (SLAC1(regular)-F, 5ʹ-GCCATTAGCGTACCTCCCAT-3ʹ; SLAC1(regular)-R, 5ʹ-GCAGATATTTTCTTCGCCAG-3ʹ). AtACT2 gene (ACT2-F, 5ʹ-GTAAGAGACATCAAGGAGAAGCTCTC-3ʹ; ACT2-R, 5ʹ-GGAGATCCACATCTGCTGGAATG-3ʹ) was used as an internal control. Aliquots of individual PCR products were resolved by agarose gel electrophoresis and visualized by ethidium bromide staining using a UV light.

## Infection assays

For *Hyaloperonospora arabidopsidis* Noco2 (*Hpa* Noco2) assay, 2-week-old Arabidopsis plants were sprayed with a suspension of 5 × 10^4^ spores ml^−1^ and monitored by lactophenol-trypan blue staining as described previously [19]. To evaluate conidiospore production, more than 42 cotyledons of Arabidopsis in each line were harvested. After trypan-blue staining, total number of conidiophores were counted in each examination under bright-field microscopy with a haemocytometer. Statistical analyses have been performed from three independent experiments.

Infection assays with *Pst* DC3000 or *Pst* DC3000 (*avrRpt2*) were performed as described previously [20] on leaves of the same leaf stage of 3-week-old plants grown under long day condition. The virulent bacterial leaf pathogen *Pst* DC3000, which causes bacterial speck disease, was grown overnight at 28ºC in NYGB liquid medium. The bacterial cells were collected by centrifugation and resuspended in 10 mM MgCl_2_ to a final density of 1.0 × 10^5^ colony-forming units (cfu) ml^–1^. For inoculation, the bacterial solution was injected into plant leaves with a syringe. The leaf discs that were excised from the infiltrated area after the indicated periods after inoculation were ground in 10 mM MgCl_2_ and serially diluted to determine the bacterial numbers. Bacterial numbers were scored at 0, 1, 2 and 3 d post inoculation. For each sample, eight leaf discs were pooled and analyzed, and the experiment was repeated six times per data point.

### Statistical analyses

Statistical Significance was determined using an unpaired Student *t*-test at *P* < 0.05.

## References

[1] Yu SP, Choi DW. Ions, cell volume, and apoptosis. Proc Natl Acad Sci USA. 2000;97:9360–9362. 10944207 10.1073/pnas.97.17.9360 PMC34029

[2] Okada Y, Shimizu T, Maeno E, et al Volume-sensitive chloride channels involved in apoptotic volume decrease and cell death. J Membr Biol. 2006;209:21–29. 16685598 10.1007/s00232-005-0836-6

[3] Kadota Y, Goh T, Tomatsu H, et al Cryptogein-induced initial events in tobacco BY-2 cells: pharmacological characterization of molecular relationship among cytosolic Ca^2+^ transients, anion efflux and production of reactive oxygen species. Plant Cell Physiol. 2004;45:160–170. 14988486 10.1093/pcp/pch020

[4] Kurusu T, Saito K, Horikoshi S, et al An S-type anion channel SLAC1 is involved in cryptogein-induced ion fluxes and modulates hypersensitive responses in tobacco BY-2 Cells. PLoS One. 2013;8:e70623. 23950973 10.1371/journal.pone.0070623 PMC3741279

[5] Barbier-Brygoo H, De Angeli A, Filleur S, et al Anion channels/transporters in plants: from molecular bases to regulatory networks. Annu Rev Plant Biol. 2011;62:25–51. 21275645 10.1146/annurev-arplant-042110-103741

[6] Kollist H, Jossier M, Laanemets K, et al Anion channels in plant cells. FEBS J. 2011;278:4277–4292. 21955597 10.1111/j.1742-4658.2011.08370.x

[7] Roelfsema MR, Hedrich R, Geiger D Anion channels: master switches of stress responses. Trends Plant Sci. 2012;17:221–229. 22381565 10.1016/j.tplants.2012.01.009

[8] Negi J, Matsuda O, Nagasawa T, et al CO_2_ regulator SLAC1 and its homologues are essential for anion homeostasis in plant cells. Nature. 2008;452:483–486. 18305482 10.1038/nature06720

[9] Vahisalu T, Kollist H, Wang YF, et al SLAC1 is required for plant guard cell S-type anion channel function in stomatal signalling. Nature. 2008;452:487–491. 18305484 10.1038/nature06608 PMC2858982

[10] Saji S, Bathula S, Kubo A, et al Disruption of a gene encoding C_4_-dicarboxylate transporter-like protein increases ozone sensitivity through deregulation of the stomatal response in *Arabidopsis thaliana*. Plant Cell Physiol. 2008;49:2–10. 18084014 10.1093/pcp/pcm174

[11] Jeworutzki E, Roelfsema MR, Anschütz U, et al Early signaling through the Arabidopsis pattern recognition receptors FLS2 and EFR involves Ca^2+^-associated opening of plasma membrane anion channels. Plant J. 2010;62:367–378. 20113440 10.1111/j.1365-313X.2010.04155.x

[12] Guzel Deger A, Scherzer S, Nuhkat M, et al Guard cell SLAC1-type anion channels mediate flagellin-induced stomatal closure. New Phytol. 2015;208:162–173. 25932909 10.1111/nph.13435 PMC4949714

[13] Koornneef A, Pieterse CM Cross talk in defense signaling. Plant Physiol. 2008;146:839–844. 18316638 10.1104/pp.107.112029 PMC2259093

[14] Spoel SH, Dong X Making sense of hormone crosstalk during plant immune responses. Cell Host Microbe. 2008;3:348–351. 18541211 10.1016/j.chom.2008.05.009

[15] Bari R, Jones JD Role of plant hormones in plant defence responses. Plant Mol Biol. 2009;69:473–488. 19083153 10.1007/s11103-008-9435-0

[16] Li B, Tester M, Gilliham M Chloride on the Move. Trends Plant Sci. 2017;22:236–248. 28081935 10.1016/j.tplants.2016.12.004

[17] Tsuda K, Katagiri F Comparing signaling mechanisms engaged in pattern-triggered and effector-triggered immunity. Curr Opin Plant Biol. 2010;13:459–465. 20471306 10.1016/j.pbi.2010.04.006

[18] Colcombet J, Mathieu Y, Peyronnet R, et al R-type anion channel activation is an essential step for ROS-dependent innate immune response in Arabidopsis suspension cells. Funct Plant Biol. 2009;36:832–843. 10.1071/FP09096 32688693

[19] Muskett PR, Kahn K, Austin MJ, et al Arabidopsis RAR1 exerts rate-limiting control of R gene-mediated defenses against multiple pathogens. Plant Cell. 2002;14:979–992. 12034891 10.1105/tpc.001040 PMC150601

[20] Tsutsui T, Nakano A, Ueda T The plant-specific RAB5 GTPase ARA6 is required for starch and sugar homeostasis in *Arabidopsis thaliana*. Plant Cell Physiol. 2015;56:1073–1083. 25713173 10.1093/pcp/pcv029

