# Supplementary material for: Involvement of S-type anion channels in disease resistance against an oomycete pathogen in Arabidopsis seedlings
Source: Commun Integr Biol. 2018 Aug 10;11(3):1–6. doi: 10.1080/19420889.2018.1495007 (PMC6132426; doi:10.1080/19420889.2018.1495007)
Supplement: Supplemental Material [file kcib-11-03-1495007-s001.docx]

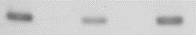

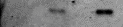

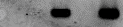

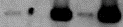


Supplemental Figure 1

*Actin 2*

*SLAC1*

(cycles)

27

21

24

27

WT

OE

**Figure S1. Expression levels of *SLAC1* mRNA in the overexpressing line of Arabidopsis.** First strand cDNA was synthesized from total RNA extracted from Arabidopsis cotyledons and amplified indicated cycles by RT-PCR as described in the materials and methods. *Actin2* cDNA was used as a control. PCR products were analyzed by agarose gel electrophoresis. WT; wild-type (Col-0), OE; *SLAC1-*overexpressor.
